# Complete genome and comparative analysis of *Streptococcus gallolyticus *subsp. *gallolyticus*, an emerging pathogen of infective endocarditis

**DOI:** 10.1186/1471-2164-12-400

**Published:** 2011-08-08

**Authors:** Dennis Hinse, Tanja Vollmer, Christian Rückert, Jochen Blom, Jörn Kalinowski, Cornelius Knabbe, Jens Dreier

**Affiliations:** 1 Institut für Laboratoriums- und Transfusionsmedizin, Herz- und Diabeteszentrum Nordrhein-Westfalen, Universitätsklinik der Ruhr-Universität Bochum, Georgstraße 11, 32545 Bad Oeynhausen, Germany; 2 Institute for Genome Research and Systems Biology, Center for Biotechnology, Universität Bielefeld, Postfach 100131, 33501 Bielefeld, Germany; 3 Bioinformatics Resource Facility, Center for Biotechnology, Universität Bielefeld, Postfach 100131, 33501 Bielefeld, Germany

## Abstract

**Background:**

*Streptococcus gallolyticus *subsp. *gallolyticus *is an important causative agent of infectious endocarditis, while the pathogenicity of this species is widely unclear. To gain insight into the pathomechanisms and the underlying genetic elements for lateral gene transfer, we sequenced the entire genome of this pathogen.

**Results:**

We sequenced the whole genome of *S. gallolyticus *subsp. *gallolyticus *strain ATCC BAA-2069, consisting of a 2,356,444 bp circular DNA molecule with a G+C-content of 37.65% and a novel 20,765 bp plasmid designated as pSGG1. Bioinformatic analysis predicted 2,309 ORFs and the presence of 80 tRNAs and 21 rRNAs in the chromosome. Furthermore, 21 ORFs were detected on the plasmid pSGG1, including tetracycline resistance genes *telL *and *tet(O/W/32/O)*. Screening of 41 *S. gallolyticus *subsp. *gallolyticus *isolates revealed one plasmid (pSGG2) homologous to pSGG1. We further predicted 21 surface proteins containing the cell wall-sorting motif LPxTG, which were shown to play a functional role in the adhesion of bacteria to host cells. In addition, we performed a whole genome comparison to the recently sequenced *S. gallolyticus *subsp. *gallolyticus *strain UCN34, revealing significant differences.

**Conclusions:**

The analysis of the whole genome sequence of *S. gallolyticus *subsp. *gallolyticus *promotes understanding of genetic factors concerning the pathogenesis and adhesion to ECM of this pathogen. For the first time we detected the presence of the mobilizable pSGG1 plasmid, which may play a functional role in lateral gene transfer and promote a selective advantage due to a tetracycline resistance.

## Background

*Streptococcus gallolyticus *subsp. *gallolyticus *(formerly known as *S. bovis *biotype I) is a gram-positive bacterium belonging to the Lancefield Group D streptococci. Over the last ten years, the classification of *S. gallolyticus *subsp. *gallolyticus *has been revised several times [1-4]. *S. bovis *was previously divided into three biotypes, designated as biotype I, biotype II/1, and biotype II/2. The majority of isolates associated with human endocarditis have been assigned to biotype I, which was recently reclassified as *Streptococcus gallolyticus *subsp. *gallolyticus *[5]. Furthermore, *S. gallolyticus *subsp. *gallolyticus *is a common member of the microflora and appears in approximately 2.5 to 15% of the gastrointestinal tract of healthy human [6,7]. It is an opportunistic human pathogen which can cause several bacterial infections, including septicemia and endocarditis. Over the last few years, the percentage of cases of endocarditis caused by group D streptococci has significantly increased [8-10]. Recently, Russel *et al. *estimated that *S. gallolyticus *subsp. *gallolyticus *is the causative agent in 24% of streptococcal endocarditis cases [11]. In addition, several studies present strong correlations between appearance of colon neoplasms and *S. gallolyticus *subsp. *gallolyticus *infection [7,12], while the underlying pathomechanisms are still unknown. Sillanpää *et al. *suggest that premalignant and malignant lesions in the intestinal tract could facilitate translocation of *S. gallolyticus *subsp. *gallolyticus *through the disrupted mucosal barrier and provide access to blood circulation [13]. Furthermore, studies have suggested a linkage between inflammation by *S. bovis *and colon carcinogenesis [14]. In addition, a variety of animal infections, such as mastitis, septicemia in poultry, lactic acidosis and infections of various ruminant animals are caused by *S. gallolyticus *subsp. *gallolyticus *[15-17]. However, the exact pathomechanisms of *S. gallolyticus *subsp. *gallolyticus *or *S. bovis *infections remain unclear.

*S. gallolyticus *subsp. *gallolyticus *shares its environment with numerous other potentially pathogenic bacteria, such as *S. agalactiae*, *Enterococcus faecalis *or others. This implies the possibility of horizontal gene transfer of antimicrobial resistance genes or genomic islands, e.g. phage-related clusters, by transposons, plasmids or phages, within the human gut or the animal rumen [18]. Several studies have reported the occurrence of competence-stimulating peptides in *S. bovis *[19]. These factors facilitate the acquisition of novel genes, resistance islands or virulence-associated regions [20], in particular when several species coexist within biofilms [21]. Recently we were able to show the capability of biofilm formation on polystyrene surfaces for *S. gallolyticus *subsp. *gallolyticus *[22]. Nevertheless, most of the mechanisms of transfer and insertion are poorly understood [23,24].

*In vitro *studies have demonstrated the adhesion and invasion of *S. gallolyticus *subsp. *gallolyticus *to extracellular matrix proteins [22,25], virulence associated proteins [13,26,27], as well as EA.hy926 or HUVEC cells [22]. Furthermore, studies have addressed biosynthesis of capsular polysaccharides [28] and fimbriae-like structures on the bacterial surface in *S. gallolyticus *subsp. *gallolyticus *[29]. It has been demonstrated that *S. gallolyticus *subsp. *gallolyticus *has 11 cell wall-anchored proteins with "microbial surface component recognizing matrix molecules" (MSCRAMM) characteristics, including a collagen-binding adhesin and proteins with similarities to pilus subunits [13].

Recently, Rusinok *et al. *published the first whole genome sequence of *S. gallolyticus *subsp. *gallolyticus *strain UCN34 and analyzed the main metabolic and cell surface features, particularly with regard to adaptation to the rumen and the virulence association of polysaccharide capsule, glucan mucopolysaccharides, different types of pili and collagen binding proteins [30].

Here we present the whole genome sequence of a not described, considerably divergent *S. gallolyticus *subsp. *gallolyticus *strain. The strain under study was the tetracycline resistant strain ATCC BAA-2069, isolated from a patient with infectious endocarditis. We demonstrate the occurrence of a previously undescribed plasmid (pSGG1) which carries genes for tetracycline resistance (*tetL, tet(O/W/32/O)*) and reveals strong sequence similarities to plasmids and chromosomes from several ruminal and gastrointestinal bacteria, indicating that pSGG1 may act as a native carrier for horizontal gene transfer.

## Results

### General genome properties

The whole genome sequence of *S. gallolyticus *subsp. *gallolyticus *was determined by pyrosequencing using the 454 GS FLX Titanium technique (Roche, Mannheim, Germany) and, after assembly of the 454 reads, remaining gaps were closed by PCR and conventional Sanger sequencing. The genome contains a 2,356,444 bp circular DNA molecule with a G+C-content of 37.65% and a previously undescribed 20,765 bp plasmid designated as pSGG1. Mapping of gene set was performed against *S. gallolyticus *subsp. *gallolyticus *genome UCN34 (GenBank Acc. No.: FN597254) [30]. Bioinformatic analysis predicted 2,309 open reading frames (ORFs), the presence of 80 tRNAs and 21 rRNAs in the chromosome, as well as 21 ORFs on the plasmid pSGG1.

**Figure 1:**
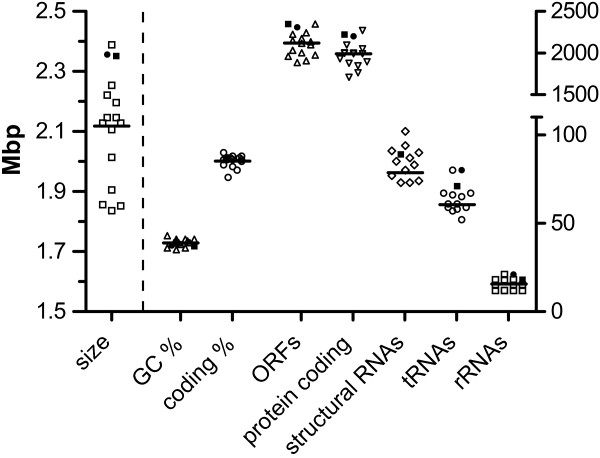
**Distribution of whole genome characteristics**. Black dot represents *S. gallolyticus *subsp. *gallolyticus *strain Isolate BAA-2069. Black square represents *S*. *gallolyticus *subsp. *gallolyticus *strain UCN-34. Symbols represent genomes of *S. agalactiae *A909, *S. dysgalactiae *subsp. *equisimilis *GGS_124, *S. equi *subsp. *equi *4047, *S. sanguinis *SK36, *S. suis *BM407, *S. uberis *0140J, *S. pyogenes *MGAS9429, *S. pneumoniae *ATCC 700669, *S. mutans *NN2025, *S. mitis *B6, *S. thermophilus *LMD-9, *S. gordonii *str. *challis *substr. CH1, *S. oralis *ATCC 35037, *S. salivarius *SK126.

The size of the BAA-2069 circular chromosome (2,356,444 bp) exceeds the average of other previously published streptococcal genomes by 12% (mean: 2.1 mb; n = 15) (Table 1, Figure 1). Direct comparison shows that only the *S. sanguinis *SK36 genome is larger (2,388,435 bp), and the G+C-content is 1.7% lower than average (range from 35.3 to 43.4%; n = 15). Altogether 2,309 ORFs were automatically annotated, which is 10% higher than the average of all complete sequenced *Streptococcus *genomes (2,107 ORFs). In direct comparison to the *S. gallolyticus *subsp. *gallolyticus *genome UCN34, the BAA-2069 genome is 5.5 kb larger (2,356,444 to 2,350,911 bp), has 70 fewer CDS (2,309 to 2,239) and contains the 20,765 bp plasmid pSGG1.

**Table 1:** Comparison of *Streptococcus*/*Enterococcus *species with *S. gallolyticus *subsp. *gallolyticus *BAA-2069

Strain	GenBank Acc No:	GC %	Coding %	Size bp	ORFs	tRNAs	rRNAs
***S. gallolyticus *subsp. *gallolyticus *BAA-2069**	FR824043	**38**	**87**	**2,356,444**	**2,309**	**80**	**21**
***S. gallolyticus *subsp. *gallolyticus *UCN34**	FN597254	37	86	2,350,911	2,349	71	18
***S. agalactiae *A909**	CP000114	35	86	2,127,839	2,136	80	21
***S. dysgalactiae *subsp. *equisimilis *GGS_124**	AP010935	39	86	2,106,340	2,174	57	15
***S. equi *subsp. *equi *4047**	FM204883	41	80	2,253,793	2,243	66	18
***S. sanguinis *SK36**	CP000387	43	88	2,388,435	2,348	61	12
***S. suis *BM407**	FM252032	41	83	2,146,229	2,118	52	12
***S. uberis *0140J**	AM946015	36	87	1,852,352	1,908	59	15
***S. pyogenes *MGAS9429**	CP000259	38	87	1,836,467	1,962	67	18
***S. pneumoniae *ATCC 700669**	FM211187	39	82	2,221,315	2,224	58	12
***S. mutans *NN2025**	AP010655	36	85	2,013,587	1,976	65	15
***S. mitis *B6**	FN568063	39	86	2,146,611	2,098	61	12
***S. thermophilus *LMD-9**	CP000419	39	76	1,856,368	2,002	67	18
***S*. *gordonii *str. *challis *substr. CH1**	CP000725	40	87	2,196,662	2,150	59	12
***S. oralis *ATCC 35037**	AEDW00000000	41	90	1,905,531	1,886	n.d.	n.d.
***S. salivarius *SK126**	ACLO00000000	40	88	2,128,332	2,034	n.d.	n.d.
***Enterococcus faecalis *V583**	AE016830	37	85	3,359,974	3,417	68	12

The sequences and annotations of chromosome and plasmid have been deposited at the NCBI GenBank (Acc. No. FR824043, FR824044).

### Comparative genomics

**Figure 2:**
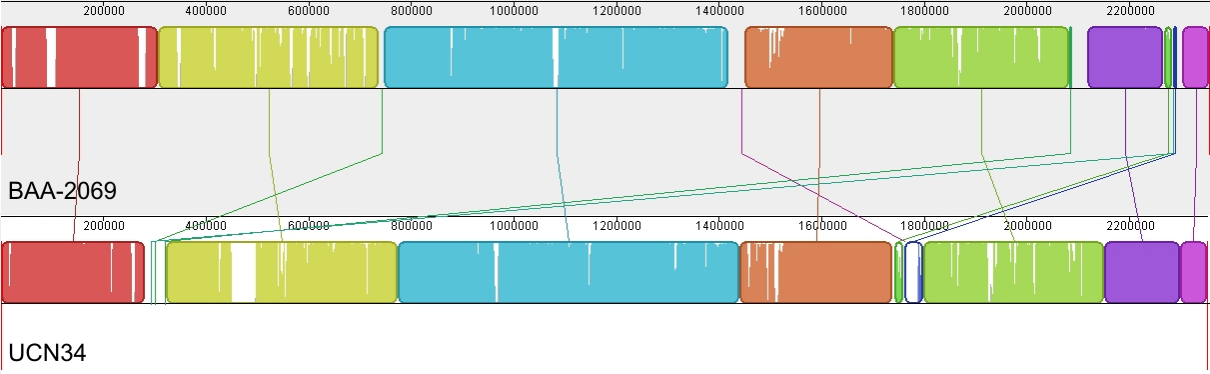
**Whole genome alignment of the two strains of *S. gallolyticus *subsp. *gallolyticus***. Representation of 13 local collinear blocks (LCB) between chromosomal sequences of the *S*. *gallolyticus *subsp. *gallolyticus *BAA-2069 and UCN34 was generated by MAUVE 2.3.1 software [31] with a minimum weight of 355. Sequence of BAA-2069 (top) is the reference against UCN34. The connecting lines between blocks indicate the location of each block in the two genomes. Each colored block represents a homologous region without rearrangements, although white areas within blocks are strain-specific.

In a direct comparison of genome BAA-2069 to UCN34, we noted various ORFs and regions inserted or deleted scattered along the genomes; nonetheless the majority of genetic information is shared by both strains. The BAA-2069 genome contains 2040 (87%) ORFs which are predicted to be common in BAA-2069 and UCN34. The arrangement of genetic information is very similar overall, based on alignment of the genomes and the synteny plot (Figure 2, Additional file 1: Figure S1). The comparison of the BAA-2069 genome with UCN34 showed about 224 kb (9.5%) of unmatched genetic information. In the UCN34 genome, 199 (9%) unique genes are present, the BAA-2069 genome contains 269 (12%) unique or weak similar genes. There are numerous strain-specific regions with functional genes originated by genetic evolution or lateral gene transfer (LGT). Due to the high number of genomic differences, we focused on genes and regions relating to putative virulence-associated functions or genes affected by habitant adaptation. All unique genes and corresponding islands calculated by EDGAR analysis are summarized in Additional file 2: Table S1 (BAA-2069) and Additional file 3: Table S2 (UCN34)

Comparison of whole chromosome sequences by MAUVE software [31] reveals an alignment consisting of 13 local collinear blocs (LCB) (Figure 2). No significant inversions or displacements of large regions between the *S. gallolyticus *subsp. *gallolyticus *genomes of BAA-2069 and UCN34 were obvious. Regions with low similarity to the corresponding genome occur frequently and their distribution is almost random, although the region from base 2,117,000 bp to the end of the genome seems to be more conserved.

### Virulence factors

The BAA-2069 genome contains a 34 kb unique insertion comprising 35 ORFs (SGGBAA2069_c20310-c20660), including the putative major cell surface adhesin *pac*. This gene is a major colonization factor in *S. mutants *[32] and may play a similar role in BAA-2069, in addition, it has a 84% similarity to a gene in UCN34 (Gallo_1675). Almost identical to this region is another 30 kb large section in the BAA-2069 genome (SGGBAA2069_c13640-c13980). Both described genetic islands could be functionally virulence-associated, comprising several proteins for cell adhesion and other virulence-determining factors.

In addition, we found a unique 23 kb genetic island in the BAA-2069 genome, coding for bacteriocin-associated genes (SGGBAA2069_c00810-c00960). This region contains genes for lanthionine biosynthesis and for a bacteriocin/lanthionine exporter orthologous to genes described in *S. mutants *and *S. ratti*. Lanthionine is a lantibiotic (bacteriocin), a unique class of peptide antibiotic substances [33]. Conducting an agar overlay experiment, we revealed an inhibited growth of *Lactococcus lactis*, resulting in a zone of clearing around BAA-2069 (data not shown).

Three genes (SGGBAA2069_ c05730, c12530, c17410) are partly homologue to hemolysin A, hemolysin III and an undefined hemolysin-like protein, although group D streptococci are usually non-hemolytic or eventually display weak alpha hemolysis. Moreover, BAA-2069 does show alpha-hemolysis on Schaedler Agar with 5% sheep blood.

The polysaccharide capsule coding region, contains 12 genes (*cpsA *- *cpsM*/SGGBAA2069_c09190 - c09300). The genes are located in a 13.5 kb region and are identical to the UCN34 genome.

### Comparison of surface proteins

We predicted 21 proteins with C-terminal LPxTG motif by *in silico *analysis. Additionally, we found orthologous or similar genes to all the proteins with MSCRAMMS characteristics described by Sillanpää *et al. *regarding the *S. gallolyticus *subsp. *gallolyticus *TX20005 genome ("*Sbs" *genes) and to genes mentioned by Rusinok *et al. *regarding the UCN34 genome ("Gallo_"-genes) [13,30]. All genes with the LPxTG motif and their best hits in related genomes are listed in Table 2.

**Table 2:** Overview and comparison of *S. gallolyticus *subsp. *gallolyticus *genes containing the LPxTG DNA motif

BAA-2069	UCN34 (Acc. No: FN597254.1)	TX20005 (Acc. No: AEEM00000000.1)	Annotation
SGGBAA2069_c01280	Gallo_0112	*Sbs10*	(*fruA*) fructan beta-fructosidase
SGGBAA2069_c05110	Gallo_0577	*Sbs16 *(64/98%)	(*cna*) collagen adhesin
SGGBAA2069_c07210	Gallo_0748	*Sbs6*	(*prtS*) lactocepin
SGGBAA2069_c10430	Gallo_1058	*Sbs2*	(*spaP*) glucan binding protein C
SGGBAA2069_c13880	Gallo_1675 (88/89%)		unknown function
SGGBAA2069_c13900			unknown function
SGGBAA2069_c14850	Gallo_1462		(*pulA*) pulluanase/glycosidase
SGGBAA2069_c15950	Gallo_1569	*Sbs11 *(100/66%)	(*fszB*) fimbrial subunit type 2
SGGBAA2069_c15960	Gallo_1570	*Sbs12 *(69/76%)	(*cna*) collagen adhesin
SGGBAA2069_c16150	Gallo_1578		(*nanA*) peptidoglycan linked protein
SGGBAA2069_c16640	Gallo_1636	*Sbs1*	(*pmrB*) major facilitator superfamily permease
SGGBAA2069_c19780	Gallo_2018		(*blpT*) putative immunity/modification protein
SGGBAA2069_c19910	Gallo_2032	*Sbs13*	(*cna*) collagen adhesin
SGGBAA2069_c19970	Gallo_2039	*Sbs14*	major pilus subunit
SGGBAA2069_c19980	Gallo_2040	*Sbs15*	(*FN1*) peptidoglycan linked protein
SGGBAA2069_c20560	Gallo_1675 (91/84%)		(*pac*) major cell-surface adhesin
SGGBAA2069_c20580			unknown function
SGGBAA2069_c21750	Gallo_2178	*Sbs7*	backbone pilus subunit
SGGBAA2069_c21760	Gallo_2179	*Acb*	(*cna*) collagen adhesin
SGGBAA2069_c22120			unknown function
SGGBAA2069_c22310	Gallo _0272 (98/95%)		(*sspA*) putative agglutinin receptor

The indicated percentage in brackets represents the query coverage to the orthologous gene and the identities revealed by blastn (two sequence alignment)

Within the analysis, we found three proteins containing the LPxTG motif carried by genomic islands specific to strain BAA-2069. The gene SGGBAA2069_c13880 and its paralog SGGBAA2069_c20560 have only very weak similarities to Gallo_1675 and code for a putative major cell surface adhesin (*pac*). The gene SGGBAA2069_c13900 and its paralog SGGBAA2069_c20580 have cell anchor characteristics but no similarities to functional genes. Furthermore, the unique protein SGGBAA2069_c22120 comprising the LPxTG motif is another gene with putative function in virulence.

### Protective elements

In comparison to *S. gallolyticus *subsp. *gallolyticus *UCN34, the BAA-2069 holds two more restriction enzyme genes. The type III enzyme *SthIR *(SGGBAA2069_c10290) is located on a 9.9 kb unique island (SGGBAA2069_c10280 - c10350), together with the corresponding restriction-methylation subunit and an integrase gene. Another type II restriction endonuclease *Eco47II *and its modification methylase is encoded on a 9.7 kb region (SGGBAA2069-c22460 - c22570).

Mentionable regions missing in BAA-2069, but present in the UCN-34 genome, are a 46 kb phage-associated region containing a putative phage-associated cell wall hydrolase. A "cluster regulatory interspaced short palindromic repeats" (CRISPR) element is sited between 1,507,890 - 1,508,913 bp and containing 16 repetitions of a 36 bp consensus sequence. Another 5.6 kb CRISPR associated region is sited at 1,515,490 - 1,516,317 bp but mostly conserved between the two strains (BAA2069 1,517,213 - 1,518,237 bp). A unique CRISPR locus for BAA-2069 is between 1,515,726 - 1,516,570 bp. Corresponding *cas *genes are for BAA-2069 SGGBAA2069_c14660 and c14670 (*cas2*), c14670 (*cas1*), respectively Gallo_1437, Gallo_1444 (*cas2*) and Gallo_1438, Gallo_1439 (cas1) for UCN34. CRISPR data of both genomes are also accessible by CRISPRs web server http://crispr.u-psud.fr.

### Genome comparison to related species

**Figure 3:**
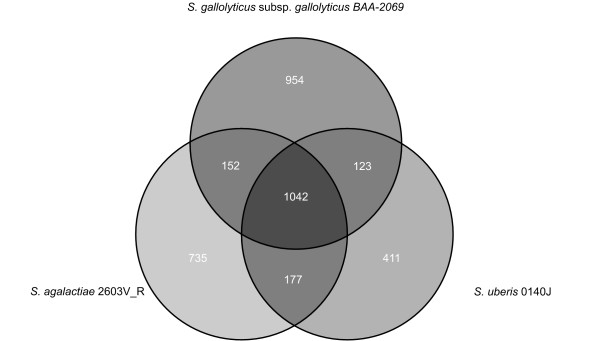
**Venn diagram of different streptococcal genomes**. Venn diagram revealed by EDGAR analysis [37]. Numbers in intersections represent the number common to two or three species. Venn diagram representing common and strain-specific genes of *S. gallolyticus *subsp. *gallolyticus *BAA-2069, *S. uberis *0140J and *S. agalactiae *2603V_R.

**Figure 4:**
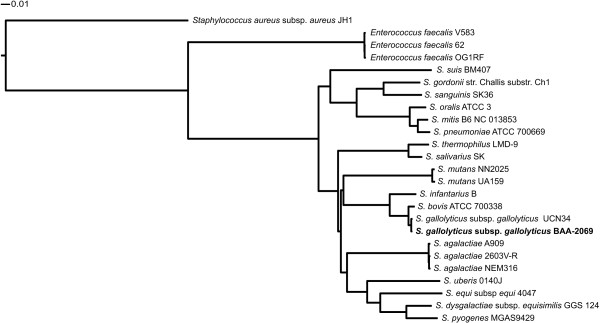
**Phylogenetic tree of different streptococcal genomes**. Tree was calculated by alignment of core genomes. Non-matching parts of the alignment were masked and subsequently removed. For further calculation details see material and methods.

To evaluate the genetic distance to related species, a direct comparison to the taxonomically most closely related species with available whole genome sequences, in particular *S. uberis *0140J and *S. agalactiae *2603V_R was conducted. The analysis revealed a core genome consisting of 1118 genes common to all three species, whereas *S. gallolyticus *subsp. *gallolyticus *BAA-2069 has 804 unique genes (Figure 3). Furthermore, we included three *Enterococcus faecalis *genomes (V583, OG1RF and 62 [34-36]). Comparison analysis revealed a set of 825 common genes, including a putative hemolysin A gene (SGGBAA2069_c05730), a fibronectin/fibrinogen binding protein (SGGBAA2069_c08170) and a sortase A gene (SGGBAA2069_c11150) which could have a possible conserved role in virulence (Additional file 4: Table S3). A complete list of common or unique ORFs in comparison to BAA-2069, considering all known *Streptococcus *genomes, is shown in Additional file 5: Table S4. Furthermore, a taxonomic analysis based on alignment of core genomes was performed (Figure 4). The calculation includes the total number of coding sequences common to all analyzed species [37]. The revealing phylogenetic tree indicates a huge genomic diversity between *S. gallolyticus *subsp. *gallolyticus *and related whole genome sequenced species.

### Plasmid

**Figure 5:**
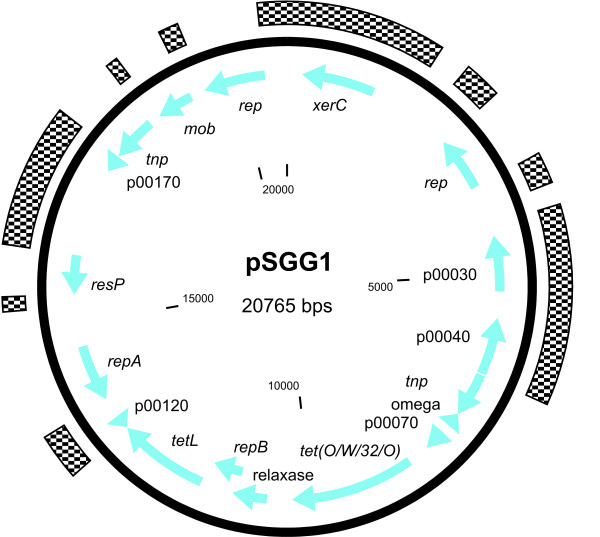
**Plasmid map of pSGG1**. Plasmid pSGG1 isolated from *S. gallolyticus *subsp. *gallolyticus *BAA-2069. Unique regions are marked by squared boxes.

**Figure 6:**
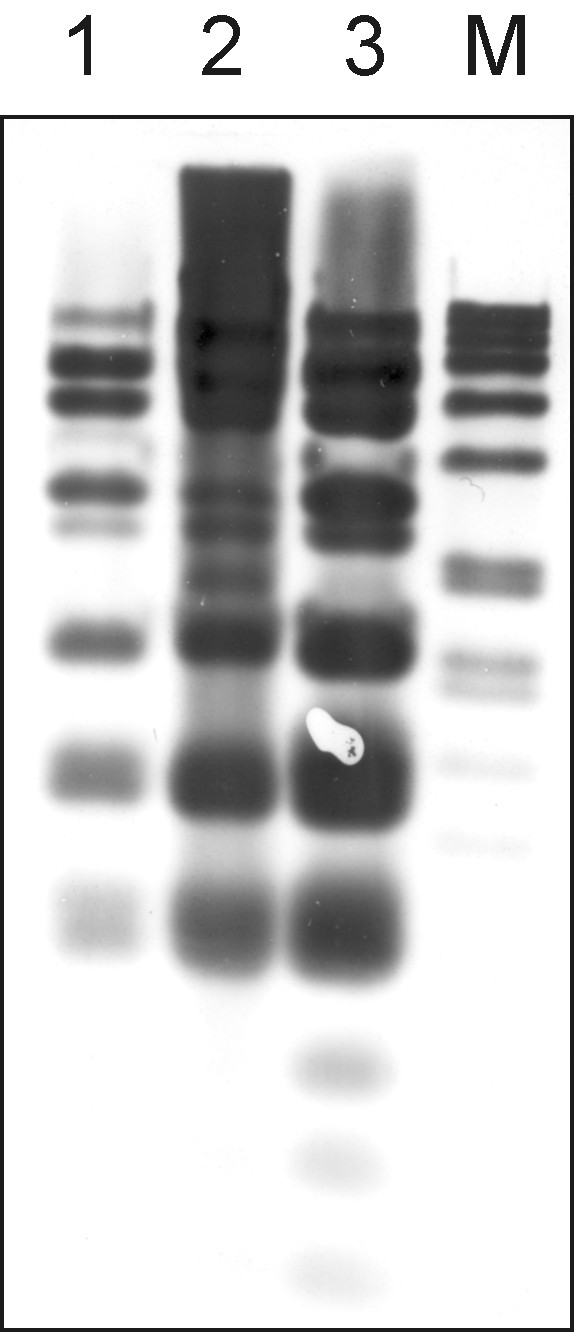
**Southern blot analysis of *BamHI*-digested plasmids from two *S. gallolyticus *subsp. *gallolyticus *strains**. Total DNA was digested with *BamHI *and hybridized against a probe consisting of DIG-11-UTP-labeled pSGG1 plasmid DNA. Lane 1: *S. gallolyticus *subsp. *gallolyticus *strain 010672 genomic DNA. Lane 2: *S. gallolyticus *subsp. *gallolyticus *strain BAA-2069 genomic DNA (positive control). Lane 3: Plasmid DNA of pSGG1. M: DIG DNA Molecular Weight Marker VII, DIG-labeled (Roche, Mannheim, Germany).

A plasmid designated as pSGG1 was identified by sequence analysis and later isolated from *S. gallolyticus *subsp. *gallolyticus *BAA-2069 (Figure 5). The plasmid pSGG1 consists of 20,765 bp and contains 21 ORFs, of which 14 genes code for proteins with similarities to sequence databases including the tetracycline resistance gene *tetL *(SGGBAA2069_p00110) and the mosaic tetracycline resistance gene *tet(O/W/32/O)*, which are common in plasmids of gram-positive pathogens. Two insertion sequence IS1216 elements and a putative resolvase and a relaxase gene were identified. The relaxase gene has similarities to plasmid pTet35 from *Campylobacter jejuni *subsp. *jejuni *81-176, which suggests its classification of the conjugative transfer system in clade MOB_P4_. Although, it is more likely that it belongs to the MOB_V _cluster, which is still ancestrally related to MOB_P _[38]. The replication is probably regulated by one of four putative *rep *elements, belonging to rep_1 superfamily (SGGBAA-2069_p00100) and rep_3 superfamily (SGGBAA-2069_p00020, p00140, p00200). The *repA *(SGGBAA-2069_p00140) element has 78% sequence identity to that of the cryptic plasmid pSBO1 isolated from *S. equinus *[39]. However, we were not able to determine the functional *rep *gene by *in silico *analysis. The plasmid pSGG1 seems to be incapable of conjugal self-transfer since it encodes no *tra *protein and only a putative resolvase, although it was not tested experimentally. Moreover, a mobilization region orthologous to a *mob *gene in *Streptococcus ferrus *was found (SGGBAA2069_p00200), which is a necessary feature for transmissible plasmids and therefore promotes the ability for LGT transfer in presence of a helper conjugative plasmid. Five ORFs were assigned to encode proteins with unknown functions and no significant sequence similarities to previously described genes exist in these cases (Figure 5). The analysis of sequence identity to other plasmids or genomes reveals a mosaic-like structure representing a high number of similarities with common habitants of the rumen or the gastrointestinal tract, including different streptococcal species as well as *Enterococcus *and others. In particular, the tetracycline resistance genes, which are very common among streptococci, are partly identical among many different plasmids and species, although no similarities in arrangement of resistance genes were observed. To evaluate the distribution of pSGG1 among strains of *S. gallolyticus *subsp. *gallolyticus *with different origin (animal, strain collections and human samples), we screened 41 strains by Southern blot hybridization analysis with a digoxygenin nick-labeled probe of pSGG1 (Figure 6). We identified and isolated a plasmid (pSGG2) mainly homologous to pSGG1 in another strain (isolate 010672), originally isolated from the blood culture of a patient with infectious endocarditis. The restriction fragment analysis of pSGG2 revealed a partially different pattern in comparison to pSGG1, indicating sequence variation between both plasmids (Additional file 6: Figure. S2). In further experiments we sequenced the pSGG2 plasmid and revealed only differences in non-coding regions (data not shown).

In order to analyze whether the frequency and phenotype of tetracycline resistance of strain BAA-2069 is coincident with the presence of pSGG1, we screened 41 *S. gallolyticus *subsp. *gallolyticus *strains for presence of *tetL *and *mob *genes by PCR. Additionally, we performed a tetracycline susceptibility test. The epidemiological cut-off for the WT of related streptococci is ≤ 1 μg/mL http://eucast.org. About 42% of strains were growth-inhibited by a tetracycline concentration between 0.5-1 μg/mL, and 95% of strains tested were unable to grow at concentrations higher than 256 μg/mL. The two strains which showed a tetracycline MIC value of 512 μg/mL carrying the pSGG plasmid, and only these were screened positively for *tetL *and *mob *genes (Additional file 7: Figure. S3).

## Discussion

The present study describes the full genome sequence of *S. gallolyticus *subsp. *gallolyticus *BAA-2069 and the comparison to related genomes in order to evaluate possible virulence-associated characteristics of this species. Previous publications have shown a significant diversity in adhesion and invasion potential for binding to endothelial host cells, as well as binding to ECM proteins *in vitro *[22]. Other studies have shown that virulence gene profiles are associated with disease [40]. Therefore genomic comparison analysis provides the basis for understanding pathogenicity.

Within whole genome comparison analysis the "pan-genome" includes a core genome containing genes present in all strains of one species. This is complemented by an individual set of genes unique to a strain or not shared by all strains. With the growing number of sequenced strains, the increasing size of the pan-genome is evidence of the genomic diversity between different isolates of a distinct species. Tettelin *et al. *have shown that in the case of *Streptococcus agalactiae *the core genome of eight strains comprises about 80% of genes of any single genome, and exploration of data reveals that the gene reservoir is immense [41], whereas in the case of *Bacillus anthracis *the number of strain-specific genes after addition of the fourth strain was zero [42]. The number of strain-specific regions in the two analyzed *S. gallolyticus *subsp. *gallolyticus *strains is, in contrast to *S. agalactiae *strains (average 7.27%, maximum ~10%), about 3.5% higher. This could be taken as a hint that, with the increasing number of sequenced strains, the pan-genome of *S. gallolyticus *subsp. *gallolyticus *is far larger by proportion. However, these data are preliminary, pending the sequencing of further *S. gallolyticus *subsp. *gallolyticus *strains.

In a direct comparison to the recently sequenced strain UCN34 [30], surprisingly many unique genes with putative virulence associated characteristics are present in each strain, which could be an indication that the pathogenicity of *S. gallolyticus *subsp. *gallolyticus *is very diverse. The majority of exclusive sequences found in the UCN34 genome are located in three large regions representing 111 kb of sequence information (53%), whereas the three largest unique regions in BAA-2069 constitute only 87 kb (39%) of strain-specific sequence and mostly consist of smaller regions. However, the tendency of virulence factors to be located within genomic islands may lead to a higher ratio of exchangeability of such genes in comparison to other regions [43]. Furthermore, additional restriction enzymes in BAA-2069 may have a function in protection against viral DNA and heritable CRISPR elements are able to mediate immunity against phages and be transmitted to other organisms by genetic transformation events [44].

Surface proteins and in particular proteins belonging to "microbial surface component recognizing matrix molecules" (MSCRAMM) were shown to play a functional role in the pathogenesis of all bacteria. Of specific interest is a group of proteins containing the C-terminal cell wall-sorting motif LPxTG, which serves as a recognition site for the membrane-associated sortase. After sortase-mediated cleavage of the protein, the polypeptide is covalently bound to the peptidoglycan of bacterial cell surface and can therefore promote the first step in bacterial adherence [45,46]. Three of the 21 predicted LPxTG motif genes are unique for BAA-2069 and further studies are required to evaluate their contribution to pathogenicity.

*In silico *analysis of genome data strongly indicated the presence of a multi-copy plasmid. The purification of plasmid DNA and further analysis of sequence data confirmed these hints and showed a localization of tetracycline resistance genes. Analysis of plasmid distribution shows only two mainly homologous plasmids in 41 strains overall. Therefore, the incidence of the pSGG plasmids among *S. gallolyticus *isolates does not seem to be widespread. The mosaic tetracycline resistance gene *tet(O/W/32/O) *is usually chromosomally located and mediates resistance by ribosome protection. It has been shown that the mosaic *tet(O/W) *genes have a higher level of resistance than the original genes [47]. This could be verified by our experimental data, showing the strains carrying the pSGG plasmid have the highest resistance levels. The *tetL *gene is generally found on plasmids and coding for a tetracycline efflux protein [48]. In contrast to the BAA-2069 strain, the tetracycline resistance of strain UCN34, mediated by *tetL *and *tetM*, was located on the chromosome and adjacent to putative plasmid and transposon Tn*916*-associated genes [30]. This indicates a strong dependence between high tetracycline resistance mediated by *tetL *and the occurrence of plasmids of the pSGG family.

Because of antibiotic treatment, gastrointestinal tract and rumen are well-known reservoirs of mobilizable antibiotic resistance genes [49]. Furthermore, the transfer of antibiotic resistance across several species and genera between commensal bacteria is well known, and habitants with a dense population and, in particular, the ability to form biofilms, are optimal for genetic transfer [50]. Especially because, *S. gallolyticus *subsp. *gallolyticus *is a commensal and facultative pathogen of animals, the intensive tetracycline treatments in animal husbandry, causes a general advantage regarding evolutionary fitness for pathogenic and natural habitants of the intestinal tract to accommodate resistance genes by LGT [51,52]. Although the plasmid pSGG1 is incapable of conjugal self-transfer, it is mobilizable by a helper conjugative plasmid. These findings suggest that it may play a functional role in LGT between different streptococcal groups and further related species. However, the detection of only two plasmids out of 41 strains is so far not evidence of LGT, but further screening of a huge variety of strains in combination with epidemiological studies should help to evaluate the role of pSGG plasmids.

## Conclusion

This study presented the analysis and comparison of the whole genome sequence of *S. gallolyticus *subsp. *gallolyticus *strain BAA-2069, a causative agent of infective endocarditis. The results promote identification of genetic factors concerning the pathogenesis and adhesion to ECM. Novel candidate genes were detected probably contributing to the pathogenicity. The comparison to *S. gallolyticus *subsp. *gallolyticus *strain UCN34 revealed significant differences concerning virulence factors, surface proteins and protective elements.

Furthermore, we detected for the first time the presence of the pSGG1 plasmid, containing 21 ORFs including mosaic tetracycline resistance genes and may play a functional role in lateral gene transfer.

## Methods

### Bacterial strains, growth conditions, nucleic acid extraction

The *S. gallolyticus *subsp. *gallolyticus *strain was isolated in 2003 at the Herz- und Diabeteszentrum Nordrhein-Westfalen from a blood culture from a 68-year-old woman with aortic heart valve endocarditis and deposited at the American Type Culture Collection (ATCC, Manassas, USA) (BAA-2069). Strain BAA-2069 was confirmed by isolation of the same strain by lesion smear test of aortic heart valve and detection in valve excision material by culture and PCR. The strain was selected because it had been defined as virulent during earlier tests [22] and shows phenotypic resistance against oxacillin, tobramycin, co-trimoxazole, colistin, metronidazole and tetracycline and intermediate resistance against gentamycin (minimal inhibitory concentration (MIC) 8 μg/mL). Isolate 010672 with plasmid pSGG2 was isolated in 2001 at the Herz- und Diabeteszentrum Nordrhein-Westfalen from a blood culture from a 62-year-old man with infectious endocarditis with no obvious connection to the origin of strain BAA-2069. For genomic DNA isolation, cells were grown for 12 h in Brain Heart Infusion Broth (BHI) (Oxoid, Hampshire, United Kingdom) at 37°C, 200 rpm. DNA extraction was performed by the Hopwood alkaline lysis method [53].

### Genome sequencing, assembly and gap closure

DNA sequencing was performed using 454 Life Science pyrosequencing technology [54], GS-FLX Titanium produced 455,842 reads of average 329 bp. The reads were assembled using Newbler V2.3, resulting in 38 contigs with 31 contigs larger than 500 bp. The large contigs obtained with 64.9× coverage served as the basis for the gap closure. Gap closure was performed by custom primer walking with long range PCR (using Phusion polymerase, New England Biolabs, Frankfurt (Main), Germany) and subsequent Sanger sequencing, resulting in 62 reads in total (IIT Biotech, Bielefeld, Germany). Long repeat structures (copies of the rrn operon and two repeats of 17.4 and 5 kbp respectively) were resolved by introducing fake reads based on the consensus sequence.

### Genome annotation

Curation and annotation of the genome were performed using the genome annotation system GenDB 2.4 [55]. Prediction of coding sequences (CDS) was accomplished using Critica [56], Glimmer [57] and Reganor [58]. All predicted ORFs were automatically submitted to similarity searches against nr, Swissprot, KEGG, InterPro, Pfam and TIGRfam databases. Putative signal peptides, transmembrane helices and nucleic acid binding domains were predicted using SignalP [59], TMHMM [60] and Helix-Turn-Helix [61], respectively. The automatic annotation of each CDS was manually checked and corrected according to the most congruent tool results.

### Genome analysis

*S. gallolyticus *subsp. *gallolyticus *BAA-2069 gene content was compared to *S. gallolyticus *subsp. *gallolyticus *UCN34, *S. agalactiae *A909, *S. dysgalactiae *subsp. *equisimilis *GGS_124, *S. equi *subsp. *equi *4047, *S. sanguinis *SK36, *S. suis *BM407, *S. uberis *0140J, *S. pyogenes *MGAS9429, *S. pneumoniae *ATCC 700669, *S. mutans *NN2025, *S. mitis *B6, *S. thermophilus *LMD-9, *S. gordonii *str. *challis *substr. CH1, *S. oralis *ATCC 35037, *S. salivarius *SK126 with EDGAR [37], which defines orthologous proteins based on bidirectional best blast hit and then calculates BLASTP score ratio values (SRV). Paralogous genes might be discarded during the analysis. For each comparison, SRV distribution was fitted with binominal or bibeta distribution with a self written R script, and a cutoff was determined at the point where the probability of belonging to one or other peak is equal. Accordingly, a general cutoff of 0.21 was used to retrieve the core genes and singletons. LPxTG-related proteins were searched by screening for [LYF]P[TSA][GANS] motif and using of an LPxTG Hidden Marcov Model for sortase substrates created by Boekhorst *et al. *[46].

### Comparison of whole chromosome sequences

Comparison of whole chromosome sequences was performed by MAUVE software using local collinear blocs (LCB). An LCB is defined as a collinear (consistent) set of multi-MUMs (exact match subsequences shared by all the considered chromosomes that appear once in each chromosome and are bordered on both sides by mismatched nucleotides). The weight (the sum of the lengths of the included multi-MUMs) of an LCB serves as a measure of confidence that it is a true orthologous region instead of a random match and is set to 355. Therefore, the ORFs or sequences between the LCBs and any regions with low similarity (shown as white in LCB) are classified as strain-specific regions.

### Calculation of phylogenetic tree

For calculation of phylogenetic tree, EDGAR was used [49]. In detail this means that, for this project comprising 25 genomes 300 core genes (orthology-cutoff 35% Score Ratio Value) of these genomes are computed. In a next step alignments of the core genes are generated using MUSCLE, non-matching parts of the alignment are masked by GBLOCKS and subsequently removed. The remaining parts of all alignments are concatenated to one huge alignment. Based on this alignment, a distance matrix is calculated using the Kimura algorithm, which is used as input for the neighbor joining method (both algorithms realized in the PHYLIP package). This leads to a phylogenetic tree, represented in newick format.

### GC skew analysis

The GC skew measures the excess of Gs by calculating the difference between the number of Gs and Cs (G-C) in a sliding window of 1000 nucleotides. The skews were cumulated to obtain the cumulative GC skew that represents the sum of the GC skews from the first to the i^th ^window.

### Plasmid screening

Screening of 41 different *S. gallolyticus *subsp. *gallolyticus *strains for presence of pSGG1 plasmid or homologs was performed by Southern-hybridization analysis in accordance with standard protocols. The probe was prepared by nick translation DIG labeling of pSGG1 referring to DIG DNA Labeling Kit (Roche Diagnostics, Mannheim, Germany) [62]. Furthermore all strains were screened for the presence of *tetL *gene by PCR using the whole genome sequence derived primer tet_f (5'-GCTATGGGAGAAGGTATCG-3') and tet_r (5'-GAGACAAACCCTGCTACTG-3'), or mob_f (5'-AAGCTGTACTTGGCTCTC-3') and mob_r (5'-CAGTGGCAGAACTATCTC-3') respectively, by standard methods.

### Nucleotide sequence accession number

Whole genome sequence of *S. gallolyticus *subsp. *gallolyticus *was deposited at GenBank (Acc. no. FR824043). Sequence of the novel designated plasmid pSGG1 was deposited with accession no. FR824044.

### Tetracycline susceptibility testing

For each strain, 200 μL BHI broth (Oxoid, Cambridge, UK) supplemented with indicated tetracycline concentration were inoculated with 1 μL of overnight culture of *S. gallolyticus *subsp. *gallolyticus *strains and cultivated in 96 well plates at 37°C. After 16 h incubation, OD _600 _was measured and growth was determined as OD _600 _> 0.2. The assay was performed in triplicate.

## Authors' contributions

DH prepared the DNA and plasmid extraction, carried out the sequence analyses, participated in the gap closure and bioinformatics analysis and wrote the manuscript. TV participated in the design of figures and helped to draft the manuscript. CR performed sequencing and carried out the sequence alignment. JB worked on bioinformatics analysis. JK participated in the design and drafted the manuscript. CK and JD conceived, designed and coordinated the study and helped to draft the manuscript. All authors have read and approved the final manuscript.

## Supplementary Material

Additional file 1 **Pairwise synteny plot of the *S. gallolyticus *subsp. *gallolyticus *BAA-2069 and UCN34 genome**. Every CDS of the first contig is checked for a reziprocal best blast hit. If one is found, the stopposition of both CDS are read from the database and used as coordinates for a dot. Click here for file

Additional file 2 **Unique genes of *S. gallolyticus *subsp. *gallolyticus *BAA-2069 in relation to *S. gallolyticus *subsp. *gallolyticus *UCN34**. Unique genes calculated by EDGAR analysis. Click here for file

Additional file 3 **Unique genes of *S. gallolyticus *subsp. *gallolyticus *UCN34 in relation to *S*. *gallolyticus *subsp. *gallolyticus *BAA-2069**. Unique genes calculated by EDGAR analysis. Click here for file

Additional file 4 **Core genome set of *S. gallolyticus *subsp. *gallolyticus *BAA-2069 and three *Enterococcus feacalis *strains**. Following strains were used for calculation by EDGAR: *E. faecalis *62 (Acc. No CP002491), *E. faecalis *OG1RF (Acc. no. CP002621) and *E. faecalis *V583 (Acc. no. NC_004668). Click here for file

Additional file 5 **Number of unique or common ORFs**. Numbers represent the common or unique ORFs in comparison to BAA-2069 and indicated species. Click here for file

Additional file 6 **Agarose gel electrophoresis of restriction fragment pattern**. Pattern were obtained with seven different enzymes, regarding plasmid pSGG2 (left lane) and pSGG1 (right lane). Ladder marker: 1 kb Ladder plus (Fermentas, St. Leon-Rot, Germany). Click here for file

Additional file 7 **Tetracycline susceptibility test**. Minimum inhibitory concentration (MIC) was determined growth in liquid cultures with indicated tetracycline concentration. Click here for file
