# Supplementary figures and images for: Complete genome and comparative analysis of Streptococcus gallolyticus subsp. gallolyticus, an emerging pathogen of infective endocarditis
Source: BMC Genomics. 2011 Aug 8;12:400. doi: 10.1186/1471-2164-12-400 (PMC3173452; doi:10.1186/1471-2164-12-400)

**Figure S1**


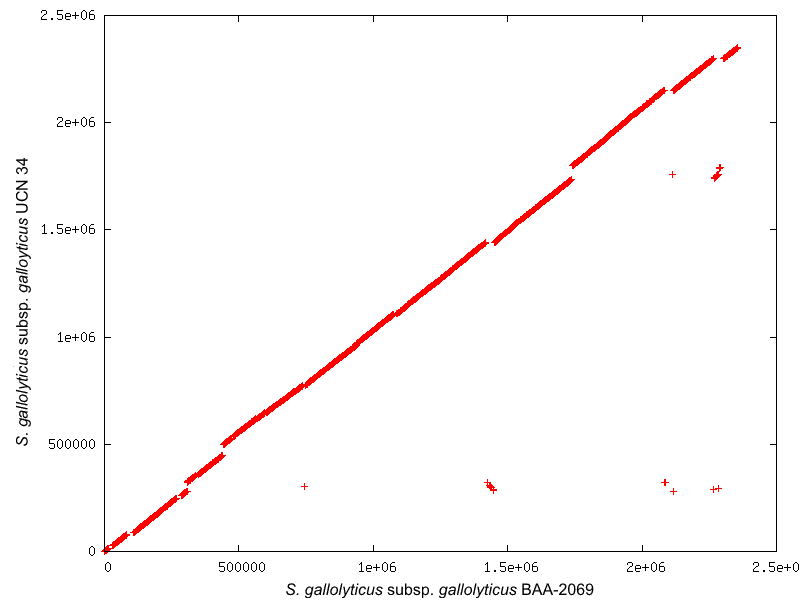


Pairwise synteny plot of the *S. gallolyticus* subsp. *gallolyticus* BAA-2069 and UCN 34 genome.

Supplement: Additional file 1 — Pairwise synteny plot of the S. gallolyticus subsp. gallolyticus BAA-2069 and UCN34 genome. Every CDS of the first contig is checked for a reziprocal best blast hit. If one is found, the stopposition of both CDS are read from the database and used as coordinates for a dot. [file 1471-2164-12-400-S1.DOC]

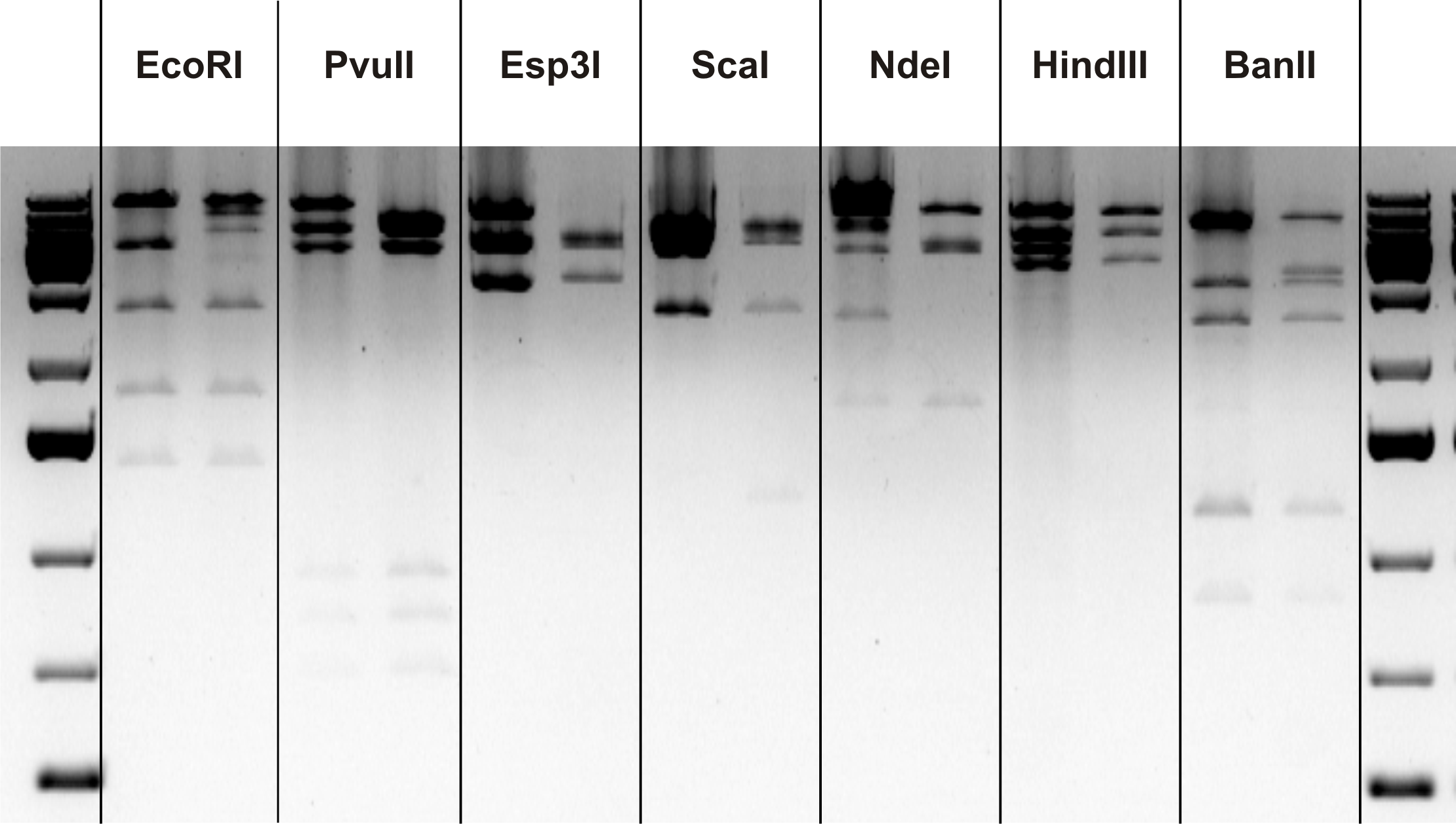

Supplement: Additional file 6 — Agarose gel electrophoresis of restriction fragment pattern. Pattern were obtained with seven different enzymes, regarding plasmid pSGG2 (left lane) and pSGG1 (right lane). Ladder marker: 1 kb Ladder plus (Fermentas, St. Leon-Rot, Germany). [file 1471-2164-12-400-S6.PNG]

Number of strains inhibited by indicated concentration

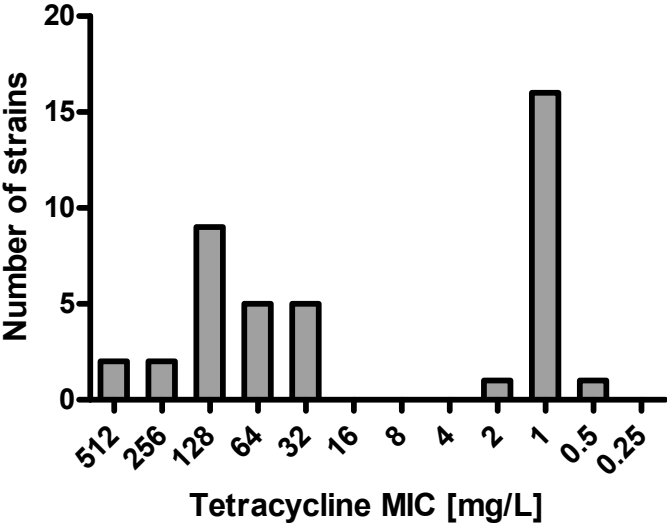

Supplement: Additional file 7 — Tetracycline susceptibility test. Minimum inhibitory concentration (MIC) was determined growth in liquid cultures with indicated tetracycline concentration. [file 1471-2164-12-400-S7.PDF]
