# Supplementary material for: Complete genome and comparative analysis of Streptococcus gallolyticus subsp. gallolyticus, an emerging pathogen of infective endocarditis
Source: BMC Genomics. 2011 Aug 8;12:400. doi: 10.1186/1471-2164-12-400 (PMC3173452; doi:10.1186/1471-2164-12-400)
Supplement: Additional file 5 — Number of unique or common ORFs. Numbers represent the common or unique ORFs in comparison to BAA-2069 and indicated species. [file 1471-2164-12-400-S5.DOC]

**Table S1**

Numbers represent the common or unique ORFs in comparison to BAA-2069 and indicated species

| **Strain** | **unique ORFs for indicated strain** | **common** | **Unique ORFs for BAA-2069** |
| --- | --- | --- | --- |
| *S. gallolyticus* subsp. *gallolyticus* UCN34 | 256 | 2029 | 308 |
| *S. mutans* NN2025 | 634 | 1257 | 1014 |
| *S. salivarius* SK126 | 724 | 1239 | 1032 |
| *S. sanguinis* SK36 | 796 | 1186 | 1085 |
| *S. gordonii* str. challis CH1 | 880 | 1169 | 1102 |
| *S. agalactiae* A909 | 1100 | 1168 | 1103 |
| *S. dysgalactiae* subsp. *equisimilis* GGS_124 | 594 | 1165 | 1106 |
| *S. suis* BM407 | 932 | 1147 | 1124 |
| *S. uberis* 0140J | 797 | 1110 | 1161 |
| *S. pneumoniae* ATCC 700669 | 884 | 1098 | 1173 |
| *S. mitis* B6 | 597 | 1094 | 1177 |
| *S. oralis* ATCC 35037 | 718 | 1093 | 1178 |
| *S. thermophilus* LMD-9 | 884 | 1082 | 1189 |
| *S. equi* subsp. *equi* 4047 | 845 | 1068 | 1203 |
| *S. pyogenes* MGAS9429 | 828 | 1036 | 1235 |
| *E. faecalis 62* | 2002 | 878 | 1414 |
| *E. faecalis OG1RF* | 1712 | 867 | 1425 |
| *E. faecalis V583* | 2245 | 856 | 1436 |
